# Impact of Maternal Valaciclovir Therapy on Early Neurodevelopment in Congenital CMV Infection: A Retrospective Pilot Study

**DOI:** 10.3390/children13040566

**Published:** 2026-04-18

**Authors:** Francesca Arcieri, Adele Vasta, Gregorio Volpe, Fabio Natale, Barbara Caravale, Daniele Di Mascio, Valentina D’Ambrosio, Michela De Cicco, Gianluca Terrin, Lucia Oliva, Costanza Prestianni, Giuseppina Liuzzi, Lucia Manganaro, Antonella Giancotti

**Affiliations:** 1 Department of Maternal and Child Health and Urological Sciences, Sapienza University of Rome, 00185 Rome, Italy; francesca.arcieri@uniroma1.it (F.A.); adele.vasta@uniroma1.it (A.V.); gregorio.volpe@uniroma1.it (G.V.); daniele.dimascio@uniroma1.it (D.D.M.); lucia.oliva@uniroma1.it (L.O.); 2 Department of Gynecology, Obstetrics and Urological Sciences, Azienda Ospedaliero-Universitaria Policlinico Umberto I, 00161 Rome, Italy; fab.natale@libero.it (F.N.); dr.valentina.dambrosio@gmail.com (V.D.); 3 Department of Developmental and Social Psychology, Sapienza University of Rome, 00185 Rome, Italy; barbara.caravale@uniroma1.it; 4 Department of Dynamic and Clinical Psychology, and Health Studies, Sapienza University of Rome, 00185 Rome, Italy; michela.decicco@uniroma1.it; 5 Department of Mother and Child Health, Policlinico Umberto I, Sapienza University, 00161 Rome, Italy; gianluca.terrin@uniroma1.it; 6 Faculty of Medicine and Psychology, Sapienza University of Rome, 00185 Rome, Italy; costanzaprestianni@gmail.com; 7 Maternal-Fetal Infection Prevention Service, National Institute for Infectious Diseases Lazzaro Spallanzani, IRCCS, 00163 Rome, Italy; giuseppina.liuzzi@inmi.it; 8 Department of Radiological, Oncological and Pathological Sciences, Sapienza University of Rome, 00185 Rome, Italy; lucia.manganaro@uniroma1.it

**Keywords:** congenital cytomegalovirus, valaciclovir, prenatal antiviral therapy, neurodevelopmental outcomes, Bayley-III, neonatal brain MRI, timing of maternal infection, early cognitive development

## Abstract

**Highlights:**

**What is the main finding?**

- Prenatal exposure to valaciclovir was associated with higher cognitive composite scores at 4–8 months compared to untreated infants with confirmed congenital CMV infection. This association persisted after adjustment for MRI findings and in sensitivity analyses accounting for symptomatic status at birth. No differences were observed in language or motor domains.

**What is the implication of the main finding?**

- These findings suggest that prenatal antiviral therapy may have an early impact on cognitive development in infants with congenital CMV infection. If confirmed in larger prospective studies, functional neurodevelopmental outcomes should be incorporated as key endpoints in the evaluation of prenatal antiviral strategies, and structured developmental surveillance should be considered for all affected infants.

**Abstract:**

**Background/Objectives:** Maternal valaciclovir therapy is increasingly used to reduce fetal viral replication in cases of primary cytomegalovirus (CMV) infection during pregnancy. However, data on its impact on early neurodevelopmental outcomes remain limited. This study aimed to evaluate the association between prenatal valaciclovir exposure and early neurodevelopment in infants with confirmed congenital CMV infection (cCMV). **Methods**: In this retrospective monocentric cohort study, 30 infants with PCR-confirmed cCMV infection were assessed at 4–8 months of age using the Bayley Scales of Infant and Toddler Development, Third Edition (Bayley-III). Infants were stratified according to prenatal exposure to maternal valaciclovir. Univariate analyses and multivariable linear regression models were performed to evaluate the association between prenatal antiviral exposure and cognitive outcome, adjusting for brain MRI findings and selected clinical variables. **Results**: Fifteen infants (50%) were exposed to prenatal valaciclovir. Exposed infants demonstrated higher cognitive composite scores compared with unexposed infants (median 105 [IQR 100–110] vs. 90 [85–110]; *p* = 0.030). In multivariable analysis, prenatal valaciclovir exposure remained significantly associated with higher cognitive scores (β = 11.89, 95% CI 2.86–20.92; *p* = 0.012), while neonatal MRI abnormalities were not independently associated with outcome. No significant differences were observed in language or motor domains. The final model explained 30% of the variance in cognitive scores (R^2^ = 0.30). **Conclusions**: Prenatal valaciclovir exposure was associated with higher cognitive composite scores after adjustment for selected covariates. Although causality cannot be inferred, these findings suggest a potential association with early neurodevelopmental outcomes and support the inclusion of functional neurodevelopmental endpoints in future prospective studies. These results should be considered exploratory and hypothesis-generating

## 1. Introduction

Congenital cytomegalovirus (cCMV) infection is the most common congenital viral infection worldwide, with an estimated global birth prevalence ranging between 0.5% and 1% of all live births, corresponding to approximately 200,000–300,000 affected newborns annually [1,2]. Global seroprevalence varies substantially across geographic and socioeconomic settings, reaching over 80–90% in certain populations [1]. cCMV represents a leading cause of non-genetic sensorineural hearing loss (SNHL), accounting for approximately 20–25% of cases in childhood [3]. Approximately 10–15% of infected neonates present clinical symptoms at birth, including intrauterine growth restriction, hepatosplenomegaly, thrombocytopenia, microcephaly, and neurological abnormalities [4,5]. Among the 85–90% of infants who are initially asymptomatic, up to 10–15% may subsequently develop long-term sequelae, particularly SNHL and neurodevelopmental impairment [4,6]. Longitudinal studies have demonstrated that neurodevelopmental sequelae extend beyond hearing loss and may involve cognitive and motor domains, particularly in infants with central nervous system (CNS) involvement [7,8]. The risk and severity of fetal infection are strongly influenced by the timing of maternal primary infection. Vertical transmission rates following primary infection range from approximately 30–40% in the first trimester to 40–70% in the third trimester [9,10]. Although transmission increases with advancing gestation, severe neurological damage is more frequently associated with first-trimester infections [9]. In fanct, first-trimester infections are linked to a higher likelihood of CNS abnormalities, including ventriculomegaly, cortical malformations, cerebellar hypoplasia, and white matter injury [11,12,13]. Neuroimaging plays a crucial role in prognostic stratification. Fetal magnetic resonance imaging (MRI) improves detection of cortical and white matter abnormalities compared with ultrasound alone [12]. Studies have shown that up to 70–80% of infants with moderate-to-severe brain MRI abnormalities develop neurodevelopmental impairment, whereas infants with normal imaging findings have significantly lower risk [6,14,15]. Advanced MRI techniques, including diffusion-weighted imaging and apparent diffusion coefficient (ADC) measurements, have further demonstrated correlations between white matter microstructural abnormalities and adverse neurodevelopmental outcomes [13]. In recent years, maternal antiviral therapy with high dose valaciclovir (8 g/day) has emerged as a potential strategy to reduce fetal viral replication in cases of primary maternal infection. A randomized controlled trial demonstrated that valaciclovir significantly reduced the rate of positive amniocentesis for CMV DNA compared with placebo (11% vs. 30%), suggesting a reduction in vertical transmission or fetal viral load [16]. Subsequent observational studies and meta-analyses have reported reductions in fetal viral load and potential improvement in imaging findings, although evidence regarding its impact on neonatal clinical presentation and long-term neurodevelopment remains heterogeneous [17,18]. Current international consensus recommendations acknowledge the potential benefit of prenatal antiviral therapy but emphasize the need for additional high-quality data on functional neurodevelopmental outcomes [19]. Beyond hearing impairment, increasing evidence indicates that cCMV may affect early cognitive and motor domains, even in infants classified as asymptomatic at birth. Prospective cohort studies have reported subtle but measurable differences in cognitive and motor performance within the first year of life, with neurodevelopmental delays observed in approximately 15–30% of infants with documented CNS involvement [8,20]. Standardized tools such as the Bayley Scales of Infant and Toddler Development allow early identification of developmental vulnerabilities and may facilitate timely intervention. Despite growing evidence regarding imaging correlations and antiviral treatment efficacy, data specifically addressing the relationship between prenatal valaciclovir exposure and early neurodevelopmental performance remain limited. Few studies have evaluated early cognitive, language, and motor outcomes within the first six months of life in treated versus untreated infants with confirmed cCMV infection. The primary aim of this study was therefore to evaluate the association between prenatal exposure to maternal valaciclovir therapy and early neurodevelopmental outcomes at approximately six months of age in infants with confirmed congenital CMV infection. Secondary objectives included assessing the influence of the trimester of maternal infection and the presence of prenatal and postnatal neuroimaging abnormalities on early cognitive, language, and motor development. By integrating epidemiology, imaging, and therapy, this study seeks to clarify the potential neuroprotective role of prenatal antiviral therapy and to refine early prognostic stratification in infants with cCMV infection.

## 2. Materials and Methods

### 2.1. Study Design and Population

This retrospective monocentric cohort study was conducted at the Prenatal Diagnosis Unit and the Child Neuropsychiatry Unit of Policlinico Umberto I, Rome, Italy. Between January 2021 and December 2024, pregnant women with suspected cytomegalovirus (CMV) infection were evaluated and prospectively followed. Data were subsequently analyzed retrospectively for the purpose of the present study.

Inclusion criteria were:

- Maternal age ≥ 18 years,
- Documented primary CMV infection occurring in the periconceptional period or during pregnancy
- Confirmed congenital CMV (cCMV) infection in the neonate by detection of CMV DNA in urine samples collected within the first 21 days of life using polymerase chain reaction (PCR).
- Availability of complete neonatal follow-up and neurodevelopmental assessment between 4 and 8 months of age using the Bayley Scales of Infant and Toddler Development.

Exclusion criteria included:

- Age < 18 years
- Non-primary CMV infection (reactivation or reinfection)
- Pregnancy termination
- Incomplete neonatal follow-up data.

After application of inclusion and exclusion criteria, 30 infants with confirmed cCMV infection were included in the final analysis.

### 2.2. Diagnostic Criteria for Maternal and Congenital CMV Infection

Maternal primary CMV infection was defined as the appearance of CMV-specific IgG antibodies in previously seronegative women (seroconversion) or by detection of CMV-specific IgM antibodies associated with low IgG avidity, consistent with recent primary infection. Congenital CMV infection was confirmed by detection of CMV DNA in urine samples collected within the first 21 days of life using polymerase chain reaction (PCR) testing according to established diagnostic criteria [19]. Infants were classified as symptomatic or asymptomatic at birth based on the presence of clinical, laboratory, or neuroimaging findings attributable to congenital CMV infection. In particular, symptomatic infection was defined by at least one of the following: petechiae, hepatosplenomegaly, jaundice, thrombocytopenia, microcephaly, growth restriction, abnormal neurological examination, abnormal cranial ultrasound or brain MRI findings, ophthalmologic abnormalities, or sensorineural hearing loss in accordance with current international consensus recommendations [19]. All neonates underwent standardized comprehensive postnatal evaluation at birth, including clinical examination, laboratory assessment, hearing screening by otoacoustic emissions, ophthalmologic examination with fundoscopy, and cranial ultrasound. In cases of abnormal otoacoustic emissions, auditory brainstem response testing was performed, whereas brain magnetic resonance imaging was reserved for infants with abnormal cranial ultrasound findings.

### 2.3. Prenatal and Postnatal Imaging

Prenatal ultrasound examinations were performed according to standardized protocols for the evaluation of suspected congenital infection, including detailed fetal anatomical assessment and targeted neurosonography, in line with the ISUOG Practice Guidelines [21]. Fetal brain magnetic resonance imaging (MRI) was performed in selected cases with sonographic suspicion of central nervous system involvement. Postnatal MRI was performed in infants presenting clinical manifestations at birth or abnormal cranial ultrasound findings and was considered the reference modality for neuroradiological assessment in this study, given its higher sensitivity in detecting and characterizing structural abnormalities [12,13,14]. Neuroradiological findings—including ventricular and cortical abnormalities, leukoencephalopathy, cerebellar abnormalities, and microcephaly—were recorded and descriptively analyzed, since the limited number of cases for each specific lesion did not allow separate correlation analyses.

### 2.4. Antiviral Therapy

#### 2.4.1. Prenatal Antiviral Therapy

Prenatal valaciclovir therapy was administered in accordance with the Agenzia Italiana del Farmaco (AIFA) determination issued on 16 December 2020 [22], which authorizes its use in cases of confirmed primary maternal CMV infection diagnosed within the first 24 weeks of gestation. Based on current evidence, prenatal valaciclovir is administered following maternal primary infection with the aim of reducing maternal viral load and lowering the risk of vertical transmission by limiting transplacental viral spread [17]. Valaciclovir was administered at a dosage of 2 g four times daily (total daily dose: 8 g/day), in accordance with AIFA recommendations. Therapy was initiated following diagnosis of maternal primary infection and continued until amniocentesis, which was performed at least 8 weeks after the onset of maternal infection and not before 20 + 1 weeks of gestation. If amniotic fluid PCR was negative, treatment was discontinued. In cases of confirmed fetal infection with evidence of mild-to-moderate fetal disease, therapy was prolonged until delivery in accordance with AIFA recommendations. In the absence of amniocentesis, treatment was continued until 26 weeks of gestation according to AIFA guidance. Amniocentesis was not performed in selected cases because of maternal refusal or late diagnosis of infection, including third-trimester maternal infections.

#### 2.4.2. Postnatal Antiviral Therapy

Postnatal antiviral management was also standardized according to contemporary neonatal recommendations [19,23]. Specifically, postnatal valganciclovir therapy was reserved for neonates with symptomatic congenital CMV infection, particularly in the presence of moderate-to-severe disease and/or central nervous system involvement. When indicated, valganciclovir was administered at the standard recommended dosage of 16 mg/kg/dose twice daily for 6 months. This approach reflects the need to balance potential therapeutic benefits against the known risks and practical burden of treatment, including hematologic toxicity—particularly neutropenia—the requirement for close laboratory monitoring, and the challenges associated with prolonged oral administration twice daily for up to 6 months during early infancy.

### 2.5. Neurodevelopmental Assessment

Neurodevelopmental assessment was conducted between 4 and 8 months of age using the Bayley Scales of Infant and Toddler Development, Third Edition (Bayley-III) [24]. An experienced child neuropsychiatrist performed neurological and developmental evaluations.

The Bayley-III is a standardized instrument designed to assess developmental functioning in children aged 1–42 months. It includes the Cognitive, Language, and Motor scales administered by trained examiners, as well as the Social-Emotional and Adaptive Behaviour scales completed by caregivers. In the present study, analyses were restricted to the Cognitive, Language, and Motor domains.

The Cognitive Scale assesses exploration, object-related behaviors, memory, attention, problem-solving, and early symbolic play. The Language Scale evaluates both receptive and expressive communication, including preverbal behaviors and early language development. The Motor Scale examines fine motor skills (e.g., grasping and visuomotor integration) and gross motor abilities (e.g., postural control and locomotion).

Based on BSID-III normative data [23], developmental expectations at approximately 6 months include, in the Cognitive domain, reaching and manipulating objects and handling multiple items; in the Language domain, responding to one’s name, interrupting activity when called, recognizing familiar words, producing vowel sounds and consonant–vowel combinations, and initiating communicative behaviors; and in the Motor domain, emerging thumb opposition, transferring objects between hands, brief independent sitting, and rolling from supine to prone.

Composite scores for the Cognitive, Language, and Motor domains were calculated (mean = 100; SD = 15) [23]. The proportion of children scoring below 85, indicative of developmental delay, was computed for each scale [23].

### 2.6. Statistical Analysis

Categorical variables are presented as number (percentage), and continuous variables are expressed as median and interquartile range (IQR). Between-group comparisons were performed using Fisher’s exact test for categorical variables and the Wilcoxon rank-sum test for continuous variables. Comparisons between infants exposed and not exposed to prenatal valaciclovir were conducted for baseline characteristics, perinatal outcomes, neuroimaging findings, hearing outcomes, and Bayley-III composite scores. Multivariable linear regression analysis was performed to evaluate the association between prenatal valaciclovir exposure and cognitive composite score, adjusting for brain MRI findings. MRI-adjusted multivariable analyses were restricted to infants with available brain MRI. For inferential analyses, brain MRI findings were analyzed dichotomously (normal vs. abnormal), with MRI classified as abnormal when at least one pathological radiological finding was identified among the predefined imaging abnormalities assessed. Variables were selected a priori based on clinical relevance. Regression coefficients (β), 95% confidence intervals (CI), and *p*-values were reported. Model fit was assessed using the coefficient of determination (R^2^). Model assumptions were evaluated graphically through residual plots and formally tested using the Shapiro–Wilk test for normality (*p* = 0.53). Homoscedasticity was assessed using the Breusch–Pagan test (*p* = 0.12). No influential outliers were identified (maximum Cook’s distance = 0.26). Analyses were conducted in complete cases, and no imputation methods were applied. A two-sided *p*-value < 0.05 was considered statistically significant. Sensitivity analyses were performed to assess the robustness of the findings. Symptomatic status at birth was included in the regression model instead of brain MRI findings to account for potential collinearity and non-uniform imaging availability. Trimester of maternal infection was assessed in baseline comparisons between groups to explore its potential role as a confounding factor. Given the retrospective and non-randomized nature of the study, potential selection bias and residual confounding should be carefully considered when interpreting the results. Statistical analyses were performed using RStudio environment (version 2026.01.0+392; Posit Software, PBC, Boston, MA, USA).

### 2.7. Ethical Approval

The study was conducted in accordance with the Declaration of Helsinki and approved by the Territorial Ethics Committee Lazio Area 1 (reference number 8194; protocol 1099/2025; approval date: 20 November 2025). Given the retrospective nature of the study, written informed consent was obtained according to institutional policy.

### 2.8. Use of Generative Artificial Intelligence

No generative artificial intelligence tools were used for data generation, statistical analysis, or interpretation of results. AI-assisted language editing was used solely for grammatical refinement.

## 3. Results

### 3.1. Study Population

**Figure 1:**
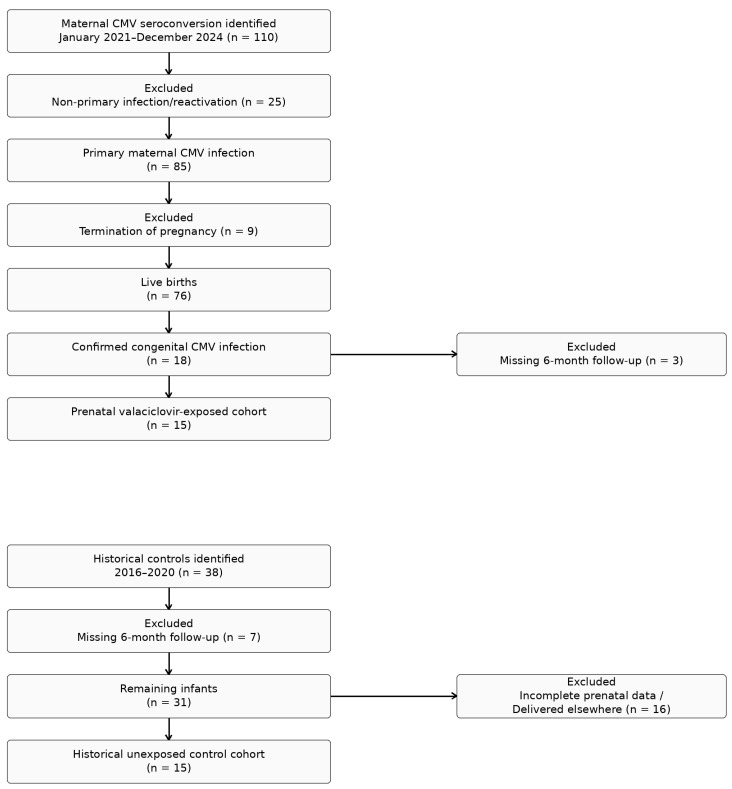
Flow Diagram of Study Population Selection.

This retrospective monocentric comparative cohort study was conducted at the Prenatal Diagnosis Unit and Child Neuropsychiatry Unit of Policlinico Umberto I, Rome, Italy. To identify the exposed cohort, all pregnant women diagnosed with maternal CMV seroconversion at our Prenatal Diagnosis Unit between January 2021 and December 2024 were screened. A total of 110 pregnancies were initially identified. After exclusion of 25 cases of non-primary infection or reactivation, 85 pregnancies with confirmed primary maternal CMV infection were included. Among these, 9 pregnancies ended in termination of pregnancy, and 76 resulted in live birth. Neonatal screening identified 18 infants with confirmed congenital CMV infection by positive urine PCR within the first 21 days of life. Three infants were excluded because 6-month neurodevelopmental follow-up was unavailable, as follow-up had been performed at external institutions. The remaining 15 infants constituted the exposed cohort. All infants in the exposed cohort had been prenatally exposed to maternal valaciclovir therapy, as prenatal antiviral treatment was routinely implemented at our institution following the Italian Medicines Agency (AIFA) determination issued in December 2020, which authorized valaciclovir use for the prevention of fetal CMV infection in cases of primary maternal infection diagnosed during pregnancy [22]. To identify the unexposed comparison group, a retrospective search was performed among infants followed at the Child Neuropsychiatry Unit prior to implementation of prenatal valaciclovir therapy. Historical cases were reviewed backward chronologically from December 2020 to May 2016. During this screening period, 38 infants with congenital CMV infection were identified. Of these, 7 were excluded because 6-month neurodevelopmental follow-up was unavailable. Among the remaining 31 infants, 16 were further excluded because prenatal obstetric records were incomplete or delivery had occurred outside our institution, precluding retrieval of complete prenatal maternal and obstetric data. The remaining 15 infants constituted the historical unexposed control cohort, allowing construction of a balanced comparative cohort with equal group sizes. All controls had documented primary maternal CMV infection, including three cases of third-trimester maternal infection. Patient recruitment and cohort selection are summarized in Figure 1.

### 3.2. Baseline Clinical and Perinatal Characteristics

For this comparative analysis, infants exposed to prenatal valaciclovir were defined as cases (exposed group), whereas infants who did not receive antiviral therapy during pregnancy were considered controls (unexposed group). Of the 30 infants included, 15 were exposed to prenatal valaciclovir and 15 were unexposed. Baseline maternal, clinical, and perinatal characteristics were comparable between groups (Table 1). The distribution of trimesters of maternal infection did not differ significantly (*p* = 0.30), and amniocentesis results were similarly distributed (*p* = 0.10). Symptomatic infection at birth occurred in 7/15 infants (46.7%) in the unexposed group and 5/15 (33.3%) in the exposed group (*p* = 0.71). In the prenatal valaciclovir-exposed group, the only infant requiring postnatal valganciclovir was a preterm neonate born at 34 weeks of gestation due to cortical abnormalities identified on neonatal neuroimaging. Mean gestational age at birth was 39.0 ± 1.2 weeks in controls and 39.1 ± 1.8 weeks in exposed infants (*p* = 0.91). Mean birth weight was 3096 g and 3364 g, respectively (*p* = 0.14), with corresponding birth weight percentiles of 36.7 and 52.4 in the unexposed and exposed groups (*p* = 0.14). Small for gestational age (<10th percentile) was observed in 3/15 infants (20%) in the unexposed group and in none of the exposed infants (*p* = 0.22) (Table 1).

### 3.3. Hearing and Ophthalmologic Outcomes

No ophthalmologic abnormalities were detected in any infant included in the study cohort. According to standard neonatal hearing screening protocols, all neonates initially underwent hearing screening by otoacoustic emissions (OAE), while auditory brainstem response (ABR) testing was performed as a second-level assessment only in infants with abnormal OAE results. Abnormal ABR findings were observed in 2/15 infants (13%) in the unexposed group and in 1/15 infant (7%) in the exposed group (*p* = 1.00). No association was observed between prenatal valaciclovir exposure and abnormal neonatal auditory outcomes (Table 2).

### 3.4. Brain MRI Findings

Brain MRI was performed in 7/15 infants (47%) in the unexposed group and 5/15 infants (33%) in the exposed group (*p* = 0.71). Among those who underwent imaging, abnormal findings were similarly distributed between groups. Ventricular abnormalities were identified in 4/7 (57%) unexposed infants and 3/5 (60%) exposed infants (*p* = 1.00). Cortical abnormalities were observed in 2/7 (29%) and 1/5 (20%) infants, respectively (*p* = 1.00). Microcephaly was detected in 3/7 (43%) unexposed infants and in none of the exposed infants (*p* = 0.20). Leukoencephalopathy and cerebellar abnormalities were each observed in 2/7 (29%) unexposed infants and in none of the exposed infants (*p* = 0.49 for both comparisons) (Table 3).

### 3.5. Univariate Analysis of Neurodevelopmental Outcomes

Neurodevelopmental assessment was performed at a median age of 6 months (IQR 6–7), with no differences between groups (*p* = 0.60). In univariate analyses, infants exposed to prenatal valaciclovir showed higher Cognitive composite scores compared with unexposed infants (median 105 [IQR 100–110] vs. 90 [85–110]; *p* = 0.030). Language and Motor composite scores did not differ between groups (Table 4).

### 3.6. Multivariate Analysis

A multivariable linear regression model was performed to evaluate the association between prenatal valaciclovir exposure and cognitive outcome, adjusting for brain MRI findings. Brain MRI was available in 12/30 infants (40%). MRI-adjusted multivariable analyses were therefore restricted to infants with available brain MRI. For inferential analyses, brain MRI findings were analyzed dichotomously (normal vs. abnormal), with MRI classified as abnormal when at least one pathological radiological finding was identified among the predefined imaging abnormalities assessed. Prenatal valaciclovir exposure remained associated with higher cognitive composite scores after adjustment (β = 11.29, *p* = 0.011). Among infants with available MRI, abnormal findings were associated with lower cognitive composite scores compared with normal MRI (β = −11.89, *p* = 0.080), although this difference did not reach statistical significance. The model explained 29% of the variance in cognitive scores (R^2^ = 0.29; model *p* = 0.028) (Table 5).

Given the limited sample size and number of available events, multivariable analyses were considered exploratory and hypothesis-generating.

Sensitivity analyses supported the robustness of the association between prenatal valaciclovir exposure and cognitive outcome. When symptomatic status at birth was included in the model instead of MRI findings, valaciclovir exposure remained associated with higher cognitive composite scores (β = 11.75, 95% CI 2.37–21.13; *p* = 0.016), while symptomatic infection was not associated with cognitive outcome.

Similarly, after adjustment for trimester of maternal infection, prenatal valaciclovir exposure remained associated with higher cognitive composite scores (β = 9.25, 95% CI 0.21–18.29; *p* = 0.045), whereas trimester of infection was not associated with outcome. No significant differences were observed between first, second, and third trimester infections (overall *p* = 0.32).

### 3.7. Additional Analysis of Valaciclovir Exposure Characteristics

Among treated pregnancies (n = 15), valaciclovir was initiated at a median gestational age of 18 weeks (IQR 13.5–23) and administered for a median duration of 9 weeks (IQR 6–13). In exploratory analyses restricted to the treated subgroup, no significant correlations were observed between gestational age at treatment initiation and cognitive composite scores (Spearman’s ρ = −0.03, *p* = 0.91), or between duration of exposure and cognitive outcome (ρ = −0.09, *p* = 0.75). Consistently, linear regression analyses showed no association between treatment timing (β = −0.03, 95% CI −0.78 to 0.72; *p* = 0.94) or duration of exposure (β = −0.18, 95% CI −0.81 to 0.44; *p* = 0.53) and cognitive composite scores.

## 4. Discussion

The role of prenatal valaciclovir in congenital CMV infection has been primarily investigated in terms of virological outcomes. The randomized controlled trial by Shahar-Nissan et al. (2020) [16] demonstrated that high-dose valaciclovir significantly reduced the rate of positive amniocentesis following primary maternal infection. Similarly, the meta-analysis by D’Antonio et al. (2023) [17] confirmed reductions in fetal infection rates and viral load, although data on long-term functional outcomes remain limited. Our findings suggest a possible association between prenatal antiviral therapy and early cognitive development in infants with congenital CMV infection. This association remained after adjustment for neonatal MRI findings and in sensitivity analyses accounting for symptomatic status at birth and trimester of maternal infection. However, these results should be interpreted with caution. Given the relatively small sample size, adjustment in multivariable models was limited to a small number of covariates, and residual confounding cannot be excluded. Notably, neither symptomatic presentation nor timing of infection was associated with cognitive performance in the adjusted analyses, suggesting that the observed association was not explained by these factors. Although causality cannot be inferred, these findings provide preliminary evidence of a possible association between prenatal antiviral therapy and early functional neurodevelopment, extending the current focus beyond virological and structural outcomes. Advanced MRI investigations by Katorza et al. (2018) [12] and more recently Barkai et al. (2024) [13] have demonstrated correlations between white matter microstructural alterations and adverse neurodevelopmental outcomes. Even in infants considered asymptomatic, early developmental assessments may be within the normal range, although subtle difficulties may still be present in specific domains [8]. These findings support the hypothesis that reducing intrauterine viral replication may help limit inflammation-related disruption of developing neural networks, even when conventional imaging does not reveal overt structural abnormalities.

In line with the observed neurodevelopmental findings, infants exposed to prenatal valaciclovir showed higher cognitive composite scores, while language and motor outcomes were similar between groups. The lack of differences in language and motor domains at 4–8 months may reflect developmental timing rather than a true absence of effect. At this age, Bayley-III cognitive scores mainly reflect early attention and emerging problem-solving abilities, whereas expressive language and more complex motor functions tend to develop later. In line with this, longitudinal studies suggest that neurodevelopmental differences may become more apparent beyond the first year of life (Dreher et al., 2014 [7]; Stoyell et al., 2024 [20]). However, these findings should be interpreted with caution, particularly given the limited sample size.

In exploratory analyses, neither gestational age at treatment initiation nor total duration of valaciclovir exposure was associated with early cognitive outcomes. The relatively late gestational age at treatment initiation observed in some treated pregnancies likely reflects the real-world timing of maternal CMV diagnosis, as primary maternal infection is frequently asymptomatic and often identified only during routine prenatal screening. Moreover, all patients were referred to a tertiary infectious disease referral center for specialist evaluation and treatment initiation, which may have further contributed to delays between diagnosis and therapy commencement. These findings suggest that the observed association between prenatal valaciclovir exposure and cognitive performance may not be driven by a dose–response or timing effect within the treatment window observed in this cohort. However, given the limited sample size of the treated subgroup, these analyses should be interpreted cautiously.

In our cohort, brain MRI was performed in a limited subset of infants, primarily those who were clinically symptomatic, resulting in non-uniform imaging availability and potential collinearity with clinical severity. Given the limited number of MRI examinations and heterogeneity of radiological abnormalities observed, a more granular MRI severity classification was not feasible. Structural abnormalities—including ventricular enlargement, cortical abnormalities, microcephaly, and white matter involvement—were observed among imaged infants, with no significant differences between groups. In multivariable analysis, abnormal MRI findings were associated with lower cognitive composite scores, although this association did not reach statistical significance. These findings are in line with recent evidence suggesting that structural imaging alone may have limited prognostic value in congenital CMV infection. In particular, Arcieri et al. [25] reported that symptomatic neonates may present with normal prenatal ultrasound and fetal MRI findings, underscoring the incomplete predictive value of imaging when considered in isolation. Overall, these findings should not be interpreted as evidence against the prognostic relevance of brain MRI in congenital CMV infection, but rather as reflecting the limited sample size and the non-systematic use of imaging in our cohort.

If confirmed in larger prospective studies, the observed association between prenatal valaciclovir exposure and improved early cognitive scores suggests that functional neurodevelopmental outcomes should be considered key endpoints in the evaluation of prenatal antiviral strategies. To date, the effectiveness of treatment has primarily been assessed in terms of virological control or reduction in structural abnormalities. However, the impact on the child’s cognitive and functional development represents a clinically meaningful outcome that may better reflect long-term benefit.

In addition, our findings highlight the importance of structured neurodevelopmental follow-up for all infants with confirmed congenital CMV infection, regardless of initial clinical presentation or imaging findings. Even infants who appear asymptomatic at birth may develop subtle developmental differences over time. Systematic monitoring is therefore essential to allow early identification of emerging vulnerabilities and timely referral for supportive or rehabilitative interventions.

### Strengths, Limitations, and Future Directions

The strengths of this study include the use of PCR-confirmed diagnosis of congenital CMV infection, standardized neurodevelopmental assessment using the Bayley-III administered by trained child neuropsychiatrists, and the application of multivariable models to explore associations while accounting for selected clinically relevant variables. However, several limitations should be considered. First, the retrospective non-randomized design introduces potential selection bias and precludes causal inference. Second, the relatively small sample size limits statistical power and restricts the number of variables that could be included in the multivariable models. As a result, adjustment was limited to a small number of covariates, and residual confounding cannot be excluded. Therefore, the observed association between prenatal valaciclovir exposure and cognitive outcome should be interpreted with caution. Third, brain MRI was performed only in a subset of infants, primarily those who were clinically symptomatic, resulting in non-uniform imaging availability and potential selection bias. Consequently, MRI-adjusted multivariable analyses were restricted to a small subgroup of infants, limiting statistical power and precluding the use of more granular MRI severity classifications. In addition, the collinearity between MRI findings and symptomatic status further constrained model specification. Finally, neurodevelopmental assessment was performed early (4–8 months), and longer follow-up is required to determine whether the observed differences persist over time and translate into clinically meaningful outcomes. Ongoing longitudinal follow-up of this cohort is currently underway, with the aim of expanding the study population and extending clinical assessment up to 24 months of age to better characterize long-term auditory and neurodevelopmental outcomes. Overall, this study should be considered exploratory and hypothesis-generating. While the findings suggest a possible association between prenatal valaciclovir exposure and early cognitive outcomes, larger prospective studies are needed to confirm these results and better define the role of antiviral therapy in congenital CMV infection. Future multicenter studies with larger cohorts and longer follow-up should aim to integrate clinical, imaging, and virological data to improve risk stratification and guide individualized management.

## 5. Conclusions

In this retrospective monocentric cohort study, prenatal valaciclovir exposure was associated with higher early cognitive composite scores after adjustment for selected covariates, while no differences were observed in language or motor domains. Neonatal MRI findings were not associated with cognitive outcome in adjusted analyses. These findings suggest a possible association between prenatal antiviral therapy and early neurodevelopmental outcomes, extending the current focus beyond virological control and structural imaging markers. If confirmed in larger prospective cohorts with longer follow-up, functional neurodevelopmental outcomes may represent a clinically meaningful endpoint in the evaluation of prenatal antiviral strategies for congenital CMV infection.

## Figures and Tables

**Table 1:** Baseline Clinical and Perinatal Characteristics According to Prenatal Valaciclovir Exposure.

Variable	Controls (No Valaciclovir) n = 15	Cases (Valaciclovir) n = 15	*p*-Value
Trimester of infection			0.30
1st trimester	7 (47%)	8 (53%)	
2nd trimester	5 (33%)	7 (47%)	
3rd trimester	3 (20%)	0 (0%)	
Amniocentesis			0.10
Not performed	11 (73%)	5 (33%)	
Negative	2 (13%)	4 (27%)	
Positive	2 (13%)	6 (40%)	
Symptomatic at birth, n (%)	7 (46.7%)	5 (33.3%)	0.71
Postnatal valganciclovir therapy, n (%)	4 (26.7%)	1 (6.7%)	0.33
Weeks of Gestational age (mean ± SD)	39.0 ± 1.2	39.1 ± 1.8	0.91
Birth weight, g (mean)	3096	3364	0.14
Birth weight percentile (mean)	36.7	52.4	0.14
Small for gestational age (<10th percentile), n (%)	3 (20%)	0 (0%)	0.22

Continuous variables are presented as mean ± standard deviation. Categorical variables are presented as number (percentage). *p*-values were calculated using Fisher’s exact test for categorical variables and Welch’s *t*-test for continuous variables, as appropriate.

**Table 2:** Neonatal Hearing Outcomes According to Prenatal Valaciclovir Exposure.

Hearing Outcome	No Valaciclovir (n = 15)	Valaciclovir (n = 15)	*p*-Value
Abnormal ABR, n (%)	2 (13%)	1 (7%)	1.00

Data are presented as number (percentage). Comparisons were performed using Fisher’s exact test.

**Table 3:** Neonatal Brain MRI Findings According to Prenatal Valaciclovir Exposure.

MRI Findings	No Valaciclovir (n = 15)	Valaciclovir (n = 15)	*p*-Value
Brain MRI performed, n (%)	7 (47%)	5 (33%)	0.71
Ventricular abnormalities *	4/7 (57%)	3/5 (60%)	1.00
Cortical abnormalities *	2/7 (29%)	1/5 (20%)	1.00
Microcephaly *	3/7 (43%)	0/5 (0%)	0.20
Leukoencephalopathy *	2/7 (29%)	0/5 (0%)	0.49
Cerebellar abnormalities *	2/7 (29%)	0/5 (0%)	0.49

* Percentages calculated among infants who underwent brain MRI. Data are presented as number (percentage). Comparisons were performed using Fisher’s exact test. Percentages for specific MRI abnormalities were calculated among infants who underwent brain MRI.

**Table 4:** Neurodevelopmental Outcomes According to Prenatal Valaciclovir Exposure.

Variable	No Valaciclovir (n = 15) (Median, IQR)	Valaciclovir (n = 15) (Median, IQR)	*p*-Value
Age at follow-up, months	6 (6–7)	6 (6–7)	0.60
Cognitive CS	90 (85–110)	105 (100–110)	0.03
Language CS	106 (89–115)	103 (94–112)	0.90
Motor CS	91 (76–107)	103 (94–110)	0.12

Values are presented as median (interquartile range). *p*-values were calculated using the Wilcoxon rank-sum test.

**Table 5:** Multivariate Linear Regression Analysis for Cognitive Composite Score.

Variable	β (Estimate)	*p*-Value
Valaciclovir (Yes vs. No)	11.29	0.011
MRI Abnormal vs. Normal	−11.89	0.080

## Data Availability

The data presented in this study are available on request from the corresponding author. The data are not publicly available due to privacy and ethical restrictions related to patient data.

## Abbreviations

The following abbreviations are used in this manuscript:

cCMV	Congenital cytomegalovirus
CMV	Cytomegalovirus
MRI	Magnetic resonance imaging
PCR	Polymerase chain reaction
IQR	Interquartile range
